# The Evolutionary Trends of Health Behaviors in Chinese Elderly and the Influencing Factors of These Trends: 2005–2014

**DOI:** 10.3390/ijerph16101687

**Published:** 2019-05-14

**Authors:** Yan Feng, Erpeng Liu, Zhang Yue, Qilin Zhang, Tiankuo Han

**Affiliations:** 1 School of Business, Guizhou Minzu University, Guiyang 550025, China; tsfy11@163.com; 2 Centre for Social Security Studies, Wuhan University, Wuhan 430072, China; qilinzhang@whu.edu.cn (Q.Z.); 2017201150077@whu.edu.cn (T.H.); 3 Institute for Social Policy Research, Zhongnan University of Economics and Law, Wuhan 430073, China; yuezhang@zuel.edu.cn

**Keywords:** Chinese elderly, health behaviors, evolution trend, influencing factors

## Abstract

As China is now facing the severe challenge of rapid population ageing, the health behaviors in Chinese elderly people are of great significance for realizing the goal of “Healthy Ageing” and the construction of a “Healthy China”. Little is known about the evolutionary trends of health behaviors in the Chinese elderly and about the factors influencing these trends; thus, the purposes of this paper are: (1) To describe the classes and evolutionary trends of health behaviors in the Chinese elderly; and (2) to explore the factors that influence the changes in the health behaviors in the elderly in China. Latent class analysis (LCA) is applied in this study to analyze the classes of health behaviors in the Chinese elderly. Growth mixture modelling (GMM) is employed to describe the evolutionary trends of the health behaviors in elderly people in China. In addition, the Bivariate analysis model is adopted to identify the influencing factors of the evolution of health behaviors. The data were derived from the Chinese Longitudinal Healthy Longevity Survey (CLHLS) from 2005 to 2014. The results reveal that the health behaviors in the Chinese elderly can be grouped into five classes: Negative, relatively negative, fair, relatively positive, and positive. Approximately 77.2% of the health behaviors in the Chinese elderly have the characteristics of “modified”, with a positive tendency. Moreover, approximately 22.8% of the health behaviors in Chinese elderly people have the characteristics of “non-modified”, with a negative tendency or remaining unchanged. The evolution of the health behaviors in the elderly in China is more affected by economic factors such as timely medical treatment during childhood, pension, occupations before the age of 60 and family income, as well as by self-rated health (SRH) and demographic characteristics such as household registration, age, and education level. Hence, various possible interventions should be made to improve the health behaviors in elderly people.

## 1. Introduction

Population ageing is one of the major challenges faced by China in this century. In 2018, the number of elderly people over the age of 60 in China had reached 249 million, accounting for 17.9% of the total population. However, in comparison with the number of elderly people in China when it first became an ageing society in 1999, the net increase is approximately 120 million [1]. It is estimated that by 2050, the number of elderly people over the age of 60 in China will exceed 450 million, with a proportion of the total population over 30% [2]. The World Health Organization points out that the increase in life expectancy rather than the improvement in quality of life is of no value, and healthy life expectancy is of more significance than lifespan [3]. While the average life expectancy of Chinese elderly people has reached 76.4 years, the healthy life expectancy is only 68.7 years [4]. Additionally, in regard to the prevalence of chronic diseases, the probability of chronic diseases in the Chinese elderly has reached 80% [5], and the Chinese elderly have the trait of “longevity but not healthy”, indicating there is a huge demand for medical and nursing resources. Long-term effects of unhealthy behaviors and lifestyle can cause diseases, especially chronic ones. Without control measures, rapid population ageing by 2030 may aggravate the burden of chronic diseases in China by at least 40% [5]. All of the facts above highlight the role of health behaviors in improving the health of elderly individuals and reducing the burden of diseases.

China has the incomparably largest elderly population in the world. The health behaviors in the Chinese elderly are of great significance in realizing the goal of “Healthy Ageing” and the construction of a “Healthy China”. Previous studies on the health behaviors in Chinese elderly people encompassed the following aspects: the status quo and determinants of health behaviors and health knowledge [6,7,8,9], the impact of health literacy and health behaviors on the health status of the elderly [10,11,12,13], health behaviors and health knowledge of the rural elderly [14,15,16,17], and the health behaviors and influencing factors of empty nester elderly people [18,19,20,21]. These studies have enriched the academic field on the health behaviors in the elderly in China, however, to be fair, their observations are only based on cross-sectional data regarding the health behaviors in the elderly and their influencing factors with a relatively limited coverage of samples in only a few provinces. Given that China’s elderly population ranks as the highest in the world with an uneven spatial distribution in urban and rural areas and different provinces, these studies cannot summarize the overall perspectives of the health behaviors in the Chinese elderly, and they are even less able to report the evolution trends and the influencing factors of health behaviors in Chinese elderly people. In fact, individuals will constantly adjust their health behaviors to be in line with external factors such as living environment, accessibility to health resources and health information technology advancement [22,23], which also offers some enlightening insights for public policies promoting health behaviors. This study will use the CLHLS data from 2005 to 2014 to reveal and classify the characteristics of the Chinese elderly’s health behaviors through the LCA. On this basis, GMM is then employed to describe the evolutionary trends of health behaviors in Chinese elderly, and a Bivariate analysis model is adopted in this study to identify the influencing factors of the evolution of health behaviors in Chinese elderly.

## 2. Materials and Methods

### 2.1. Data

CLHLS provides the reliable data used in this study. The survey was jointly carried out by the Center for Healthy Aging and Development Studies at Peking University and Duke University. As the largest survey around the world, currently, in terms of health and longevity, it conducted a baseline survey in 1998, followed by six wave surveys in 2000, 2002, 2005, 2008, 2011, and 2014 from 23 random sample areas in 31 provincial administrative units of China (Beijing, Tianjin, Hebei, Shanxi, Liaoning, Jilin, Heilongjiang, Shanghai, Jiangsu, Zhejiang, Anhui, Fujian, Jiangxi, Shandong, Henan, Hubei, Hunan, Guangdong, Guangxi, Chongqing, Sichuan, Shanxi, and Hainan), all over the Eastern, middle and Western regions as well as Northeast China. After randomly selecting approximately 50% of the counties in the above 23 provincial administrative units, the survey had covered approximately 85% of China’s total population, which, approximately, can be seen as a random sampling survey among the national elderly [24]. CLHLS consists of the health status and influencing factors of the elderly from physiology, soma and cognition, including the demographic characteristics, lifestyles, health services, social participation, economic status, and family characteristics, etc. These data provide rich information and data quality has been widely tested and recognized [25,26]. More detailed information on CLHLS can be found at: http://www.icpsr.umich.edu/icpsrweb/NACDA/studies/36179.

The respondents of the CLHLS baseline survey were elderly people aged 80 years and over, and the age range after 2002 was adjusted to 65 years and over. Previous studies have found that the elderly aged 105 and below could validly fill out the questionnaire, which is generally consistent with developed countries, but the elderly aged 106 and over may underperform [27]. Therefore, in order to ensure our research quality, this study eliminated the samples of people aged 106 years and over from the data analysis. Since respondents differentiated in age range and other specific characteristics between the surveys in 1998, 2000, 2002, and the last four follow-ups, this study excluded the data from the previous 3 waves and only used CLHLS data from 2005, 2008, 2011 and 2014. For the data merging of the four waves, ID (identity code) was the only matching condition, and data from 2723 elderly people aged 65 to 105 who participated in the survey in 10 years were retained. Then, balanced panel data was constructed accordingly.

### 2.2. Variables

#### 2.2.1. Manifest Variables of Health Behaviors

In light of the design of the CLHLS questionnaire on health behaviors in the elderly, manifest variables of health behaviors selected in this paper were grouped into five dimensions: diet, exercise, social activities, smoking, alcohol consumption, including 11 health behavior variables (see Table 1). The following principles were followed when selecting specific health behaviors: first, fully consider the connotation of health behaviors and use frequency of manifest variables of health behaviors in prior studies [7,28,29], then select the existing manifest variables of health behaviors which are representative and have been fully verified by research. Second, in terms of data processing, the manifest variables related to health behaviors in the CLHLS questionnaire were included in the LCA for exploratory analysis, and the health behaviors variables with low correlation between manifest variables and latent variables were excluded. What must be noted is that the purpose of the manifest variables of health behaviors is to find the latent variables of health behaviors through LCA rather than generating new variables by simple calculations based on specific health behaviors.

#### 2.2.2. Influencing Factors of the Evolution of Health Behaviors

Based on previous studies [6,14,25,30], influencing factors of the evolution of the elderly’s health behaviors selected mainly consists of social security and services, SRH, family characteristics, and demographic characteristics of the elderly. Descriptive statistics of variables are shown in Table 2.

### 2.3. Methods

#### 2.3.1. Latent Class Analysis (LCA)

The LCA is used to identify latent classes of manifest variables of the health behaviors in the Chinese elderly, with the primary goal being to determine the individual’s potential characteristics according to an individual’s manifest variables of health behaviors and obtain the proportion of different types of people. Then, different intervention strategies will be adopted for different subgroups [31,32]. Many variables in studies cannot be directly measured, and these are called “latent variables”. Relative to the manifest variables which can be directly measured by instruments or methods such as scales or experiments, latent variables are often the characteristics or abstract concepts originating from manifest variables, which may be not be able to be directly measured, but have to be reflected or classified based on data or characteristics of manifest variables. As for the applicable conditions, model parameters or implementation steps of the LCA, this paper will not be redundant. Studies in this field can be observed in previous studies [31,32,33,34].

This analysis utilized LatentGold4.5 (Statistical Innovations Inc., Belmont, MA, USA) to identify the latent classes of health behavior variables of the Chinese elderly using the following procedures: (1) Gradually increase the number of manifest health behavior variables, starting by calculating the Null Model with only one manifest variable of health behavior, and then compare the fit indexes until the optimal model is found. (2) On the basis of determining the optimal model, the tendency of each class is determined based on the conditional probability. The greater the conditional probability is, the greater the probability that the latent class will be selected for the manifest variables, which indicates a more noticeable tendency. (3) The characteristics of the latent classes are then induced according to the conditional probability of the manifest variables in order to determine the latent class of health behaviors and name them. (4) Lastly, the health behaviors in each sample of the elderly are classified by calculating the Posterior probability with the purpose of inferring the latent class to which the sample of the elderly belongs.

#### 2.3.2. Growth Mixture Modelling (GMM)

Given that the data types in this study were used as panel data, after using LCA to classify the types of health behaviors in the elderly, GMM was used to describe the evolutionary trends of health behaviors in the elderly in the 10 years from 2005 to 2014. This model can investigate not only the heterogeneous growth of the elderly but also the variability of individual development trends within the group. In the setting of the fit indexes of the model, since the setting of the GMM starts from the Null Model with only one class and then gradually increases the number of classes, Bayesian information criterion (BIC) is chosen as the main index to determine whether the model is optimally fit, and the BIC correlates negatively with the quality of fitness [31,32]. At the same time, Log Likelihood (LL), Akaike Information Criterion (AIC), Entropy, LMR (Lo-Mendell-Rubin), and Bootstrap Likelihood Ratio Test (BLRT) were simultaneously reported for reference.

#### 2.3.3. Bivariate Analysis Model

After using the LCA and GMM to describe the latent classes of the elderly’s health behaviors and their evolutionary trends, the evolutionary results of the elderly health behaviors were treated as a bivariate variable (modified = 1, non-modified = 0), used as explanatory variables. The Bivariate analysis model was employed to research the impact of social security and services, SRH, family characteristics and demographic characteristics on the evolution of health behaviors in the elderly.

## 3. Results

### 3.1. LCA Results of Health Behaviors in Chinese Elderly

In this study, LatentGold4.5 was used to conduct an exploratory LCA of manifest variables of the health behaviors in CLHLS in 2005, 2008, 2011 and 2014 and to determine whether there are latent class variables with a significant explanatory power among the manifest variables of health behaviors in all waves. Based on manifest variables of the health behaviors in each wave, six latent class models were constructed from a Null model with only one class. The LCA fit indexes of each wave survey (see Table 3) demonstrates that, among the manifest health behaviors in all four waves, there exists the latent variable of health behaviors in the Chinese elderly. In addition, the BIC value was used as the main fit index to determine model fitness. Relative to models in all the waves, Model 5 is the best model.

According to the conditional probability (horizontally or vertically) of health behavior variables in LCA, and its possible impact directions (“+” or “−“) on the health of the elderly (see Appendix A Table A1), the health behaviors in each wave can be grouped into five classes: Negative, relatively negative, fair, relatively positive, and positive. Table 4 presents the latent class probability for Chinese elderly’s health behaviors in each wave survey.

### 3.2. Evolution Results of Health Behaviors in Chinese Elderly

The evolution of health behaviors generally has three trends: better, unchanged, and worse. Interactive analysis was first used to describe the results of the health behaviors in the elderly in our study. As shown in Table 5, from 2005 to 2014, the health behaviors in the Chinese elderly are generally changing in a more positive trend. However, it is acknowledged that to determine the evolutionary trends of the health behaviors in the elderly based only on the classes at the beginning and end of the wave may be biased, as this approach does not consider the adjustment of health behaviors over the course of the decade. Moreover, this practice could also not clearly present the evolutionary trends of elderly’s health behaviors. In view of this point, based on the 4 wave panel data over ten years of elderly’s health behaviors, three GMMs were established by using Mplus7.0 (https://www.statmodel.com/verhistory.shtml). The fit indexes of each model are shown in Table 6. Except for the Null Model with only one class in the three models, the BIC of Model 2 is the smallest, thus it is the optimal model. While Model 3 classifies the evolution of elderly’s health behaviors into three classes, one of the health behaviors’ evolution classes accounts for only 1.9% of the sample size (approximately 52 people), and the evolutionary trend is not highly differentiated from other classes. Considering all of these factors, Model 2 was the optimal model.

The estimated values of the intercept factors and the slope factors corresponding to GMM are presented in Table 7. The mean of the intercept factors and the slope factors was −3.261 (*p* < 0.01) and 0.278 (*p* < 0.01) in class 1, 0 and −0.130 (*p* < 0.01) in class 2. By comparing the mean of the slope factors of the two classes, it can be found that the evolutionary trend of the health behaviors type represented by class 1 is increasing and that of class 2 is decreasing. This study has uniformly adjusted the health behaviors variables generated by the LCA. That is, the higher the value represented, the better the health behavior type. It can be inferred that in class 1, health behaviors over 10 years have been optimized, and health behaviors in class 2 remained unchanged. As the results of the health behaviors’ evolution can generally be divided into three classes of better, unchanged, and worse, it can be concluded that class 2 represents a trend towards worse or unchanged over the past decade.

The essence of behavior modification is the use of theories of learning to deal with the individual behaviors, emotions and other aspects for treatment. The goal of behavior modification includes not only eliminating the specific bad behaviors of the individual but also cultivating and developing good behaviors [35,36]. Considering all the theoretical and quantitative significance of the evolutionary outcomes of the two classes of health behaviors, it can be inferred that Class 1 represents a positive type of evolution defined as “modified”, which accounts for approximately 77.2% of the total sample size; Class 2 stands for the class of health behaviors whose evolutionary trend is negative or unchanged, which is defined as “non-modified”, accounting for approximately 22.8% of the total sample size.

**Figure 1:**
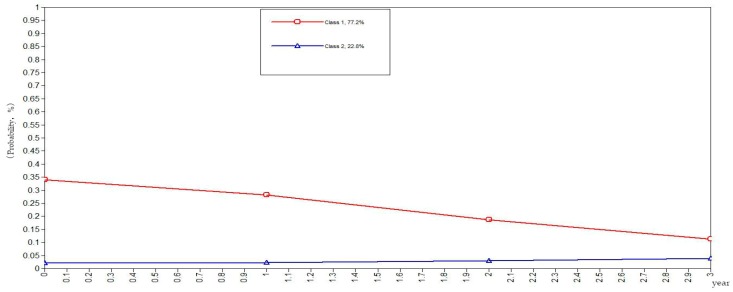
Evolution trends of the elderly with negative health behaviors.

**Figure 2:**
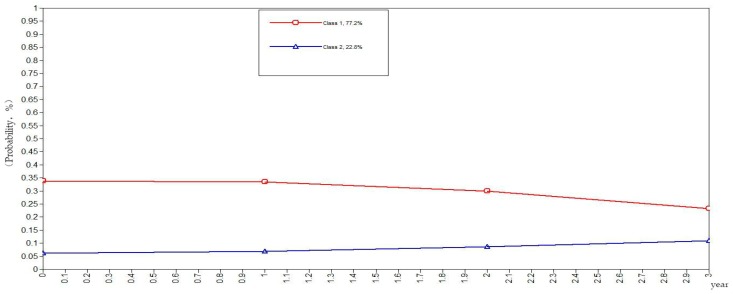
Evolution trends of the elderly with relatively negative health behaviors.

**Figure 3:**
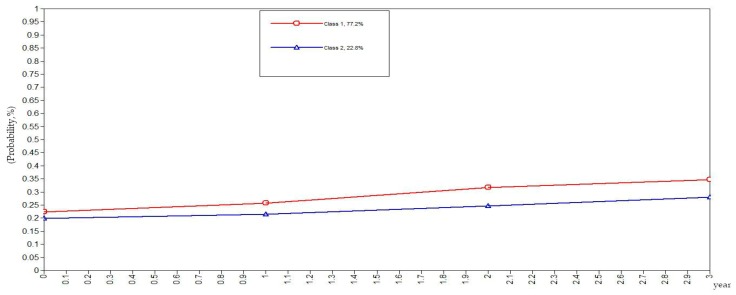
Evolution trends of the elderly with fair health behaviors.

**Figure 4:**
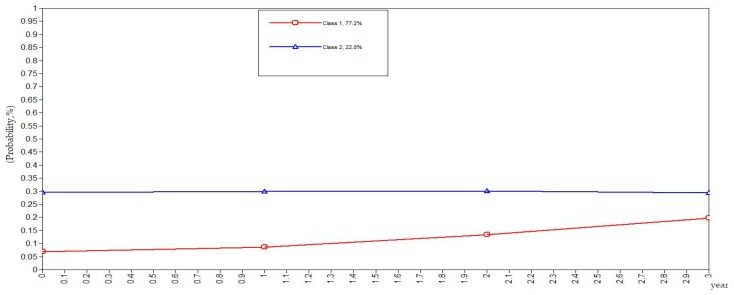
Evolution trends of the elderly with relatively positive health behaviors.

**Figure 5:**
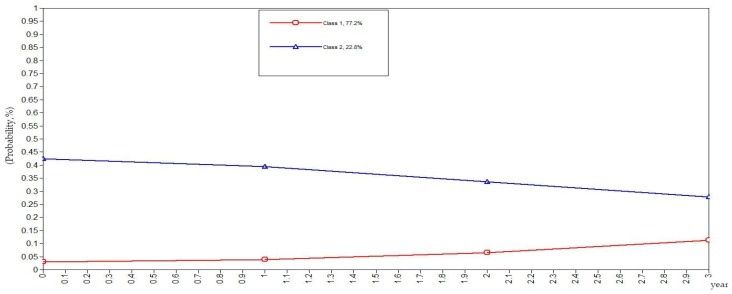
Evolution trends of the elderly with positive health behaviors.

To be more rigorous and scientific, this study set the classes of health behaviors in the elderly as categorical variables in the GMM. The GMM of class variables did not yield the evolutionary trend of the sample population but rather yielded the evolutionary trends of the subclasses as illustrated in Figure 1, Figure 2, Figure 3, Figure 4 and Figure 5. The ordinate in the graph represents the estimated probability value of health behavior modification, which ranges from 0–1. The abscissa represents different time periods. CLHLS data of the four waves in 2005, 2008, 2011, and 2014 are selected for this study. There are three time periods, and the abscissa value range is 0–3. It can be observed from Figure 1 that the elderly with negative health behaviors have a lower estimated probability of behavior modification as their age increases, while the estimated probability of non-modification is slightly increased. What can be gathered from Figure 2 is that the modifications of the elderly with relatively negative health behaviors is similar to that of the elderly with negative health behaviors. From Figure 3, it can be found that the estimated probability of behavior modification with increasing age is higher for those with fair health behaviors, although the probability of those with non-modified behaviors is also slightly elevated. However, in this class, the estimated value of probability for modified behaviors is always higher than that of the non-modified ones. It is revealed in Figure 4 that the behavior modification probability value of the elderly with relatively positive healthy behaviors is higher, and the value of the elderly with non-modified behaviors is relatively stable. The estimated probability value of behavior modification in this class is always lower than the non-modified one. From Figure 5, it is reflected that the estimated probability value of behavior modification for positive health behaviors gets higher with age, and the value of non-modified behaviors is decreasing.

In general, elderly people with positive health behaviors have a better tendency for behavior modification. Among the various classes of health behaviors, the older the elderly with negative and relatively negative health behaviors is, the lower the probability of behavior modification will be. In addition, the elderly with fair, relatively positive and positive health behaviors would pay more attention to optimizing health behaviors as their age increases.

### 3.3. Influencing Factors of the Evolution of the Elderly’s Health Behaviors

In this study, the evolution results of the elderly’s health behaviors were processed as Bivariate variables (modified or non-modified). The Bivariate analysis model was used to estimate social health and services, SRH, family characteristics and individual characteristics of the evolution of health behaviors in the Chinese elderly, analyses were performed using Stata (Stata version 13.0 for Windows, StataCorp LP, College Station, TX, USA). The results are shown in Table 8. In general, as the variables continue to increase, the explanatory power of the model is gradually increasing. Specifically, the R^2^ of Model I is 0.180, suggesting that social security and services can explain the variance of 18.0% of the evolution of the elderly’s health behaviors. The R^2^ of Model II is 0.189, and SRH can explain only the variance of 0.9% of the evolution of health behaviors. Model III’s R^2^ is 0.201, and family characteristics can explain the variance of 1.2% of the evolution of the elderly’s health behaviors. Model IV’s R^2^ is 0.253, so the individual characteristics can explain the variance of 5.2% of the evolution of the elderly’s health behaviors.

What can be inferred from the regression results of these variables is that the timely treatment of illness in childhood has a significant negative effect on behavior modifications of the elderly. The probability of behavior modification of the elderly who received timely medical treatment in childhood is significantly lower than those who did not. The former is approximately 26.3–39.9% lower than the latter (*p* < 0.05, *p* < 0.001), indicating that the accessibility of medical services in childhood significantly reduced the elderly’s emphasis on health behaviors and the sense of modification for risky health behaviors. The probability of behavior modification of elderly people with pensions is significantly lower than that of those without pensions. The former is approximately 63.9–88.2% lower than the latter (*p* < 0.001), indicating that pension plays a role as a reverse incentive in the evolution of health behaviors. The probability of behavior modification of the elderly whose self-rated their own health as “unhealthy” is significantly higher than those who chose “very healthy”; the former’s probability is 2.868–2.989 (*p* < 0.001) times higher than the latter’s. With one additional unit in family annual income (taking the natural logarithm), the probability of behavior modification of the elderly decreased by 17.8–26.9% (*p* < 0.001).

The probability of behavior modification of the elderly increased by 3.1% with one year of ageing (*p* < 0.001). The probability of behavior modification of the elderly was reduced by 6.5% (*p* < 0.001) with a one-year-advancement in attaining education. The urban/county/rural distinction in the evolution of health behaviors in the elderly is also very significant, and the probability of behavior modification of the elderly is notably lower in those living in urban settings. The probability of behavior modification of the elderly in the country is 1.813 times (*p* < 0.001) higher than those in urban areas, and the probability of behavior modification in the rural elderly is also noticeably higher than the urban elderly with the former being 3.06 times (*p* < 0.001) higher. The differences of evolution are also distinct between occupational types; the probability of behavior modification of the elderly who were engaged in the public sector before the age of 60 was 39.5% lower than those who worked as commercial, service or industrial staff before the age of 60 (*p* < 0.01). The probability of behavior modification of the elderly engaged in agricultural works before the age of 60 is notably higher than that of the elderly who were engaged in commercial, service, or industrial jobs before the age of 60, the former being 1.525 times higher than that of the latter (*p* < 0.05). Hence, it can be inferred that there is a low probability of behavior modification of the elderly with relatively high-level occupations before the age of 60 and between the occupational level and the optimization of health behaviors exists a reverse correlation.

## 4. Discussion

LCA is widely adopted in the study of health behaviors. GMM can investigate the heterogeneity of the group and describe the trends within the group and is considered to be the most commonly used and most influential model for dealing with group heterogeneous growth. This study, as far as we know, is the first study to reflect the evolutionary trends of the health behaviors in the elderly in China based on LCA and GMM through large sample panel data, with the purpose of providing new evidence on the evolution of health behaviors of the Chinese elderly. We also further explored the influencing factors of that evolution trends, and some inspiring conclusions were drawn.

The BIC value was used as the main fit index for LCA. The Entropy value and AIC value of the model were simultaneously referenced. The health behaviors in Chinese elderly people in 2005–2014 can be categorized into five classes: Negative, relatively negative, fair, relatively positive, and positive. The evolution results of the elderly’s health behaviors were accordingly divided into two types: “modified” and “non-modified” through GMM. As seen from the results, 77.2% of Chinese elderly people reported a healthier trend in the 10 years from 2005–2014 while a total of 22.8% of elderly people who reported no modification in their health behaviors over the past 10 years were unchanged or even worse.

More specifically, with the increasing of age, the elderly with negative and relatively negative health behaviors find it more difficult to optimize their health behaviors, which implies that the unhealthy behaviors are strongly irreversible and extensible, thus timely and early intervention is essential. For the elderly with fair healthy behaviors, the estimated probability of behavior modification gets higher with age increasing, but the probability of unmodified behaviors are also slightly increased, indicating that the behavior optimization process of the elderly with fair health behaviors is more complicated. The elderly with general health behaviors still have a large probability of behavior optimization in that a probability of this type is always higher than the non-modified one. The relatively positive health behaviors and positive health behaviors in the elderly have a higher probability of behavior modification, and the possibility of behavior optimization of the elderly with non-modified behaviors is elevating.

The analysis of the influencing factors on the evolution of health behaviors in the Chinese elderly shows that economic factors have a significant reverse effect on the process of the elderly’s behavior modification. These factors include the timely treatment of disease during childhood, pensions, higher occupational level before the age of 60, higher family income, and urban household registration, all of which largely represent an adequate economic situation of the elderly. All of the above factors will reduce the probability of the elderly’s modification of their health behaviors. Specifically, because the childhoods of the elderly in these samples was concentrated between the 1950s and 1960s, when the Chinese public health and medical service system had just gotten started, the ability to enjoy proper medical services represents a higher social class and good economic condition. The availability of a pension and pension levels are closely related to occupations before the age of 60, and whether they have urban household registration or not. China’s pension system has obvious regional and occupational features, and its main coverage is in urban areas. The occupational groups covered are mainly concentrated within authority unit or institutions servants, state-owned enterprise employees and workers with urban household registration. These occupational categories are all in the upper and middle classes in Chinese occupational system, which brings higher social classes, social capital and economic income, and guarantees that they can get more adequate pensions after the age of 60. This conclusion has also been confirmed in previous studies [37,38]. However, high-income Chinese families have higher consumption levels of junk food, tobacco, and alcohol. Chinese elderly people with higher socioeconomic status tend to have more negative behavior trends. In addition, the lower the age and the less education they receive, the higher the probability of health behavior modification of the elderly will be, in a negative correlation.

## 5. Conclusions

Health behaviors play a significant role in determining the health status of individuals. Based on the above analysis, this study indicates that the improvement of the health behaviors in the Chinese elderly needs to be performed through the following three modes. The first is to conduct various forms of health education by extending health knowledge and enhancing self-discipline in the health behaviors in the elderly. The focus of this action should be on elderly groups with greater likelihood of health behavior modification, such as the rural elderly, the oldest of the elderly, and the elderly with less education. Furthermore, we should strengthen the construction of the monitoring system for the elderly’s health status and take measures to intervene as soon as possible for the elderly with unhealthy behaviors. The data analysis shows that the elderly with poor SRH are more likely to modify their behaviors. Based on this, a health monitoring system covering all of the elderly needs to be established to help the elderly confirm their health status in time and constrain their unhealthy behaviors. Lastly, considering that economic factors (pension, family income, etc.) have a negative impact on health behavior modification in the elderly, it is necessary to enhance the impact of family members, primary health care institutions and other groups (such as community service agency, social work group, etc.) on urging the elderly to control and modify risky health behaviors. Family members should promptly identify the unhealthy behaviors in the elderly in their daily lives and urge them to promptly modify such behaviors. Primary health care service institutions should also provide continuous service and guidance on daily diet, exercise, and health management for the elderly.

## 6. Future Areas of Research

Constrained by the CLHLS data structure, we failed to provide wider and more detailed information about the health behaviors in the elderly, such as health knowledge development, sleeping habits, occupational health protection, safe sexual behavior, etc. These factors are also vital to studies on health behavior. Thus, future research would lay more emphasis on a full-scale set of measurement indices of elderly health behaviors, expanding the sample size and ensuring a more balanced sample distribution to carry out more comprehensive and scientific measurements of health behaviors in the Chinese elderly. At the same time, the health outcomes of elderly’s health behaviors and their changes are worthy of further study. For example, what are the differences in activities of daily living, instrumental activities of daily living, cognitive ability, and frailty index among the elderly with different health behaviors?

## Figures and Tables

**Table 1:** Descriptive statistics for manifest variables of health behaviors.

Manifest Variables of Health Behaviors		Measurement	2005 (%)	2008 (%)	2011 (%)	2014 (%)
Diet	Staple Food Types	rice = 1	68.42	68.49	67.99	66.58
		whole grains = 2	3.20	3.82	4.77	6.10
		flour = 3	14.95	12.60	13.33	12.27
		half rice and half flour = 4	12.89	14.95	13.66	14.47
		others = 5	0.55	0.15	0.44	0.59
	Fresh Vegetables	rarely or never eat = 1	1.47	1.40	2.31	3.89
		sometimes eat = 2	8.96	7.09	6.06	6.79
		often eat = 3	33.13	23.47	25.96	28.53
		eat almost every day = 4	56.45	68.05	65.66	60.78
	Tea	rarely or never drink = 1	48.44	45.98	58.24	63.28
		sometimes drink = 2	19.43	15.98	14.40	15.79
		drink almost every day = 3	32.13	38.05	27.36	20.93
	Fresh Fruits	rarely or never eat = 1	23.83	20.97	26.99	28.83
		sometimes eat = 2	39.88	39.22	35.81	35.70
		often eat = 3	25.01	26.77	21.59	21.45
		eat almost every day = 4	11.27	13.04	15.61	14.03
	Pickles	eat almost every day = 1	27.87	31.95	38.74	39.96
		sometimes eat = 2	33.46	45.61	42.16	44.47
		rarely or never eat = 3	38.67	22.44	19.10	15.57
Exercise	Physical Activity	rarely or never = 1	52.81	44.88	40.25	56.22
		past or present = 2	24.72	36.43	40.54	16.23
		past and present = 3	22.48	18.69	19.21	27.54
Social activities	Outdoor Activity	rarely or never = 1	20.24	23.50	25.71	33.79
		sometimes = 2	6.57	5.07	5.69	4.30
		monthly = 3	4.81	4.33	2.20	5.25
		weekly = 4	12.52	12.60	11.64	9.92
		daily = 5	55.86	54.50	54.76	46.75
	Playing Cards or Mahjong	rarely or never = 1	74.15	77.27	79.36	78.96
		sometimes = 2	5.29	4.52	4.33	6.10
		monthly = 3	3.82	3.05	1.98	2.39
		weekly = 4	8.26	5.62	5.66	5.21
		daily = 5	8.48	9.55	8.67	7.34
	Organized Social Activities	rarely or never = 1	79.29	82.08	78.99	81.60
		sometimes = 2	10.43	7.31	10.76	9.14
		monthly = 3	3.31	4.30	3.01	2.94
		weekly = 4	2.79	2.35	2.94	2.28
		daily = 5	4.19	3.97	4.30	4.04
Smoking	Smoking	past and present = 1	22.81	18.51	18.07	16.78
		past or present = 2	16.45	20.60	20.31	18.29
		rarely or never = 3	60.74	60.89	61.62	64.93
Alcohol Consumption	Alcohol Consumption	past and present = 1	19.10	13.40	13.88	14.51
		past or present = 2	17.96	23.32	22.51	14.32
		rarely or never = 3	62.95	63.28	63.61	71.17

**Table 2:** Description of influencing factors of the evolution of health behaviors.

Variable		Definition	Mean	Std. Dev.	*p*-Value
Social Security and Services	Community Health Knowledge Services	yes = 1, no = 0	0.393	0.488	0.787
	Community Medical Services	yes = 1, no = 0	0.290	0.454	1.000
	Timely medical treatment during childhood	yes = 1, no = 0	0.418	0.493	<0.000
	Timely medical treatment during 60-year-old	yes = 1, no = 0	0.907	0.291	<0.000
	Pension	yes = 1, no = 0	0.244	0.430	<0.000
	Health Insurance	enrolled = 1, unenrolled = 0	0.710	0.454	<0.000
SRH	Self-rated Health	very unhealthy = 1, other = 0	0.015	0.120	0.936
		unhealthy = 1, other = 0	0.111	0.314	1.000
		basically healthy = 1, other = 0	0.313	0.464	0.173
		healthy = 1, other = 0	0.431	0.495	0.351
		very healthy = 1, other = 0	0.131	0.337	<0.000
Family Characteristics	Living Arrangements	living with family = 1, other = 0	0.860	0.347	0.133
		living alone = 1, other = 0	0.129	0.335	0.948
		living in nursing homes = 1, other = 0	0.011	0.103	<0.060
	Family Income	natural logarithm after continuous measurements	8.022	1.059	<0.000
Personal Characteristics	Age	continuous measurements (year)	74.587	7.445	1.000
	Education Attainment	continuous measurements (year)	2.684	3.712	<0.000
	Gender	Male = 1, female = 0	0.473	0.499	<0.000
	Marital Status	having spouse = 1, no spouse = 0	0.584	0.493	<0.000
	Household Register	city = 1, other = 0	0.176	0.381	0.133
		county = 1, other = 0	0.192	0.394	0.948
		village = 1, other = 0	0.632	0.482	<0.060
	Occupation before 60	professional or technical staff = 1, other = 0	0.064	0.245	<0.000
		public sector staff = 1, other = 0	0.046	0.210	<0.000
		commercial, service or industrial staff = 1, other = 0	0.165	0.371	<0.000
		farmers = 1, other = 0	0.654	0.476	1.000
		Domestic labour/unemployed = 1, other = 0	0.072	0.258	0.805

**Table 3:** Latent class analysis fit indexes.

Model Class	BIC	AIC	Entropy	LL	Npar	df
2005						
Model 1	60,894.3581	60,705.2309	1.0000	19,092.1759	32	2693
Model 2	59,966.0066	59,705.9568	0.5863	18,068.9018	44	2681
Model 3	59,502.3513	59,171.3787	0.6319	17,510.3237	56	2669
Model 4	59,441.3072	59,039.4120	0.6456	17,354.3570	68	2657
Model 5	59,408.4455	58,935.6276	0.6073	17,226.5726	80	2645
Model 6	59,436.9065	58,893.1660	0.5919	17,160.1110	92	2633
2008						
Model 1	59,066.1312	58,877.0628	1.0000	17,633.1189	32	2688
Model 2	58,148.6061	57,888.6370	0.5713	16,620.6931	44	2676
Model 3	57,678.5509	57,347.6812	0.6262	16,055.7373	56	2664
Model 4	57,614.4777	57,212.7073	0.5925	15,896.7635	68	2652
Model 5	57,607.6464	57,134.9755	0.5901	15,795.0316	80	2640
Model 6	57,608.8999	57,065.3283	0.5717	15,701.3844	92	2628
2011						
Model 1	58,444.2999	58,255.4558	1.0000	17,296.1445	32	2669
Model 2	57,571.9083	57,312.2477	0.5476	16,328.9364	44	2657
Model 3	57,196.7022	56,866.2251	0.6006	15,858.9137	56	2645
Model 4	57,121.7644	56,720.4707	0.5887	15,689.1594	68	2633
Model 5	57,087.1854	56,615.0752	0.5871	15,559.7639	80	2621
Model 6	57,090.0723	56,547.1456	0.5859	15,467.8343	92	2609
2014						
Model 1	55,120.7346	54,932.8526	1.0000	16,078.0258	32	2589
Model 2	53,958.8978	53,700.5601	0.6339	14,821.7333	44	2577
Model 3	53,683.4328	53,354.6394	0.6433	14,451.8126	56	2565
Model 4	53,545.8533	53,146.6041	0.6320	14,219.7773	68	2553
Model 5	53,506.5868	53,036.8819	0.6401	14,086.0551	80	2541
Model 6	53,538.1354	52,997.9748	0.6101	14,023.1480	92	2529

Note: Npar represent number of free parameters, df represents degree of freedom.

**Table 4:** Latent class probability of health behaviors in Chinese Elderly (%).

Name	Negative	Relatively Negative	Fair	Relatively Positive	Positive
Latent class probability in 2005	30.1908	23.2208	24.3580	12.6559	9.5745
Latent class probability in 2008	35.8061	17.2237	18.8028	17.7745	10.2828
Latent class probability in 2011	29.7099	18.5090	23.3199	19.8311	7.8223
Latent class probability in 2014	33.7491	19.0756	22.0103	14.7836	6.5297

**Table 5:** Evolution of health behaviors in the Chinese elderly 2005–2014 (%).

Health Behaviors in 2005	Health Behaviors in 2014				
	Negative	Relatively Negative	Fair	Relatively Positive	Positive
Negative	5.36	45.31	18.39	26.92	4.02
Relatively Negative	9.64	6.64	63.35	6.00	14.38
Fair	6.78	8.73	51.66	6.17	26.66
Relatively positive	5.52	25.87	15.99	43.31	9.30
Positive	3.45	14.56	35	26.82	26.82

**Table 6:** GMM fit indexes.

Model	LL	AIC	BIC	aBIC	Entropy	LMR	BLRT	Latent Class Probability
Model 1	−16,481.337	32,980.675	33,033.860	33,005.265	-	-	-	1
Model 2	−16,396.243	32,816.486	32,887.400	32,849.272	0.698	0.0000	0.0000	0.772/0.228
Model 3	−16,386.108	32,802.215	32,890.858	32,843.198	0.769	0.0000	0.0001	0.740/0.019/0.242

Note: aBIC represent Adjusted BIC.

**Table 7:** GMM results of the Chinese elderly’s health behaviors.

Class		Estimate	Std. Err	Estimate/Std. Err	*p*-Value
Class 1	Intercept Mean Value	−3.261	0.099	−32.832	0.000
	Slope Mean Value	0.278	0.022	12.460	0.000
Class 2	Intercept Mean Value	0.000	0.000	999.000	999.000
	Slope Man Value	−0.130	0.025	−5.213	0.000
Between-class	Intercept Variance	0.169	0.065	2.592	0.010
	Slope Variance	0.000	0.000	0.012	0.991
	Intercept 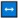 Slope	0.000	0.007	0.020	0.984

**Table 8:** Bivariate regression of the influencing factors of elderly’s health behaviors evolution.

Variable	Model I		Model II		Model III		Model IV	
	b	Exp(b)	b	Exp(b)	b	Exp(b)	b	Exp(b)
Community Provides Health Knowledge Services	0.045	1.046	0.029	1.030	0.052	1.054	0.039	1.040
Community Provides Medical Services	0.140	1.150	0.158	1.171	0.158	1.171	0.012	1.012
Illness Timely Treated in Childhood	−0.509 ***	0.601	−0.492 ***	0.611	−0.471 ***	0.625	−0.305 **	0.737
Illness Timely Treated in 60-year-old	−0.397	0.672	−0.289	0.749	−0.187	0.830	−0.083	0.921
Pension	−2.136 ***	0.118	−2.132 ***	0.119	−1.920 ***	0.147	−1.018 ***	0.361
Health Insurance	−0.172	0.842	−0.164	0.849	−0.196	0.822	−0.141	0.868
Self-rated Health (Very healthy = 0)								
very unhealthy			1.040 *	2.829	0.945	2.573	0.673	1.960
unhealthy			1.054 ***	2.868	0.999 ***	2.714	1.095 ***	2.989
basically healthy			0.205	1.228	0.138	1.147	0.111	111.7
healthy			0.163	1.177	0.131	1.140	0.048	26.64
Living Arrangements (Living in nursing home = 0)								
living with families					0.824	2.279	0.872	2.391
living alone					0.844	2.325	0.600	1.822
Family Income					−0.313 ***	0.731	−0.184 ***	0.832
Age							0.030 ***	1.031
Education Attainment							−0.067 ***	0.935
Male							0.121	1.128
Having Spouse							−0.127	0.880
Household registration (City = 0)								
County							0.595 ***	1.813
Village							1.118 ***	3.060
Occupational (Commercial, service or industrial = 0)								
professional or technical							−0.201	0.818
public sector							−0.503 *	0.605
Farmers							0.422 **	1.525
Other							0.242	1.274
Cons	2.658 ***	14.267	2.307 ***	10.043	4.751 ***	115.706	0.348	1.416
Log likelihood	−1058.950		−1047.659		−1031.925		−965.026	
Chi-square Statistics	466.000		488.590		520.050		653.850	
Pseudo R^2^	0.180		0.189		0.201		0.253	
Sample Size	2723		2723		2723		2723	

Note: b represent coefficient of interpretation; *, **, *** indicate significance at levels of 10%, 5%, and 1%, respectively.

## Abbreviations

The following abbreviations are used in this manuscript:

AIC	Akaike’s Information Criterion
BIC	Bayesian Information Criterion
BLRT	Bootstrap Likelihood Ratio Test
CLHLS	Chinese Longitudinal Healthy Longevity Survey
GMM	Growth Mixture Modeling
LCA	Latent Class Analysis
LL	Log Likelihood
LMR	Lo-Mendell-Rubin
SRH	Self-rated Health

## Appendix A

**Table A1:** Directions of each health behavior on elderly health.

Manifest Variables of Health Behaviors	2005					2008					2011					2014				
	Class 1	Class 2	Class 3	Class 4	Class 5	Class 1	Class 2	Class 3	Class 4	Class 5	Class 1	Class 2	Class 3	Class 4	Class 5	Class 1	Class 2	Class 3	Class 4	Class 5
Staple Food Types	−	−	−	+	+	−	−	−	−	+	−	−	−	−	+	+	−	−	−	+
Fresh Vegetables	−	−	+	+	+	−	−	+	+	+	−	+	+	+	+	−	+	+	+	+
Tea	+	−	−	+	+	−	−	+	+	−	−	+	−	+	+	−	+	−	+	−
Fresh Fruits	−	−	−	−	+	−	−	−	+	+	−	−	−	−	+	−	−	−	−	+
Pickles	+	+	+	+	+	+	+	+	+	−	−	+	+	+	+	−	+	+	+	+
Physical Activity	−	−	+	+	+	−	−	+	−	+	−	−	−	+	+	−	−	−	+	+
Outdoor Activity	−	−	+	+	+	−	−	+	+	+	−	−	+	+	+	−	+	+	+	+
Playing Cards or Mahjong	−	−	−	+	−	−	−	+	−	+	−	−	−	+	−	−	−	−	+	−
Organized Social Activities	−	−	−	+	+	−	−	−	−	+	−	−	−	+	+	−	−	−	+	+
Smoking	−	+	+	−	+	−	+	−	+	+	+	−	+	−	+	+	−	+	−	+
Alcohol Consumption	−	+	+	−	+	−	+	−	+	+	+	−	+	−	+	+	−	+	−	+
Total	(2+, 9−)	(3+, 8−)	(6+, 5−)	(8+, 3−)	(10+,1−)	(1+,10−)	(3+, 8−)	(6+, 5−)	(7+, 4−)	(9+, 2−)	(2+, 9−)	(3+, 8−)	(5+, 6−)	(7+, 4−)	(10+,1−)	(3+, 8−)	(4+, 7−)	(5+, 6−)	(7+, 4−)	(9+, 2−)
Class	N	RN	F	RP	P	N	RN	F	RP	P	N	RN	F	RP	P	N	RN	F	RP	P
LCP (%)	30.1908	23.2208	24.3580	12.6559	9.5745	35.8061	18.8028	17.2237	17.7745	10.2828	29.7099	23.3199	18.5090	19.8311	7.8223	33.7491	22.0103	19.0756	14.7836	6.5297

Note: “+” or “-“ represent the impact direction of behavior on the health of the elderly; N = Negative, RN = Relatively Negative, F = Fair, RP = Relatively Positive, P = Positive; LCP = Latent Class Probability.
